# Illuminating retinoid flux in the neurosensory retina

**DOI:** 10.1172/JCI201863

**Published:** 2026-02-02

**Authors:** Ala Moshiri, Akrit Sodhi

**Affiliations:** 1 Department of Ophthalmology and Vision Science, School of Medicine, University of California, Davis, Sacramento, California, USA.; 2 Wilmer Eye Institute, Johns Hopkins University School of Medicine, Baltimore, Maryland, USA.

## Abstract

The retinoid chromophore 11-*cis*-retinal triggers an intracellular cascade known as phototransduction that converts light into electrochemical signals. Enzymatic regeneration of 11-*cis*-retinal sustains vision, prevents the buildup of toxic byproducts, and is supported largely by the retinal pigmented epithelium. Directly visualizing rapidly changing retinoid intermediates in patients with inherited retinal diseases (IRDs) could provide essential therapeutic insights. In this issue, Engfer et al. introduced a groundbreaking strategy using the mouse retina as a genetically malleable model for the mammalian eye. Using cell-specific expression of lecithin:retinol acyltransferase to trap mobile retinols, they mapped the availability of 11-*cis*- and all-*trans*-retinoids within different retinal compartments under normal and diseased conditions. Their findings elucidate retinoid distribution in the retina and highlight important differences between mouse and human Müller glia. Here, we contextualize these advances within decades of research defining the visual cycle and retinoid biology, outlining the profound implications for therapeutic development for IRDs.

Vitamin A (all-*trans*-retinol) and its derivatives, collectively known as retinoids, are essential for vision. Specifically, the retinoid 11-*cis*-retinal is a chromophore that captures incoming photons. The capture of a photon alters 11-*cis*-retinal to all-*trans*-retinal and facilitates activation of opsin molecules in photoreceptor cells, triggering an intracellular signal cascade known as phototransduction. This process results in alteration of potential across the photoreceptor cell membrane, resulting in conversion of light to electrochemical changes in the vision processing areas of the central nervous system.

The vertebrate visual cycle maintains photoreceptor function by continuously regenerating 11-cis-retinal after it is bleached in the photoreceptor outer segment (OS) and reconfigured to all-*trans*-retinal during phototransduction. The spent chromophore must be reisomerized (recycled) to sustain vision and, importantly, to prevent the buildup of toxic byproducts that could damage photoreceptors. This enzymatic regeneration process relies heavily on support cells, particularly cells of the retinal pigmented epithelium (RPE). In a multistep process involving movement between photoreceptor OSs and RPE cells, all-*trans*-retinal is converted to all-*trans*-retinol, then to all-*trans*-retinyl ester (all-*trans*-RE), then to 11-*cis*-retinol and, finally, back to the 11-*cis*-retinal configuration before it is returned to the photoreceptor OS (Figure 1A).

Despite the visual cycle’s fundamental importance and biochemical precision, our understanding of how retinoids move and are processed within specific neural retinal compartments remains incomplete. In particular, we lack detailed knowledge of retinoid dynamics within the photoreceptor inner segment (IS), the supportive Müller glial cells (MGs), and the signal-transmitting retinal ganglion cells (RGCs). This critical knowledge gap limits our ability to fully understand inherited retinal disease (IRDs) that arise from defects in retinoid-handling proteins and has impeded efforts to develop rationally designed therapies aimed at modulating or correcting the visual cycle.

In the present issue, Engfer et al. have introduced an elegant experimental system that addresses this challenge (1). Using cell type–specific expression of lecithin:retinol acyltransferase (LRAT), a key visual cycle enzyme normally confined to the RPE, the authors created a biological “retinoid trap.” LRAT converts retinols into retinyl esters (REs), which are stable and fluorescently detectable, allowing for direct visualization of retinoid pools using two-photon imaging and quantification using HPLC. The distribution of different REs can serve as an indicator of the presence of free retinol in the various retinal compartments examined. Using this clever strategy, the authors provide compelling insights into the relative abundance, cellular origin, and movement of retinoids within different retinal compartments (summarized in Figure 1B).

## Building upon the bedrock of visual cycle discoveries

This work builds on decades of foundational studies defining the enzymatic steps of the visual cycle and the distribution of retinoid-binding proteins. The canonical visual cycle pathway, established through seminal biochemical and genetic analyses by Palczewski, Kiser, Travis, and colleagues, describes the reduction of all-*trans*-retinal in photoreceptors, its esterification by LRAT, and its isomerization by the enzyme RPE65 to generate 11-*cis*-retinol within the RPE (2–4). Additional work by Redmond and others has elucidated the role of RPE65 in the isomerization step, while studies by Cornwall, Koutalos, and Swaroop have characterized the kinetics of chromophore turnover, toxic retinoid byproducts, and genetic perturbations that lead to photoreceptor vulnerability (5–8). Yet even with these advances, the spatial dynamics of retinoids within the neural retina have remained technically inaccessible because of their low abundance and rapid turnover. Engfer et al. have taken a critical step toward closing this methodological gap, revealing features of retinoid distribution that align with but also extend prior biochemical predictions (1).

## Monitoring retinoid trafficking in the mammalian retina

In Engfer et al.’s report, expression of LRAT in photoreceptors resulted in accumulation of both 11-*cis*- and all-*trans*-REs within photoreceptor IS, demonstrating that both retinoid species are accessible to this compartment (1). Notably, the authors showed substantial accumulation of 11-*cis*-retinyl palmitate and stearate, implying a steady-state presence of 11-*cis*-retinol within IS membranes under physiological conditions. This observation is consistent with earlier hypotheses that a fraction of 11-*cis*-retinol supplied from the RPE diffuses into the IS before pigment regeneration, a concept that was difficult to test with traditional biochemical tools. The study also shows that IS-localized REs decrease in mice deficient in cellular retinaldehyde-binding protein (CRALBP), a retinoid-binding chaperone expressed in MGs and RPE, where it plays a critical role in the respective visual cycles by carrying regenerated 11-*cis*-retinal to the photoreceptors. This observation supports a role for MG-associated CRALBP in supplying 11-*cis*-retinal to the outer retina, consistent with prior biochemical models of CRALBP-mediated retinoid shuttling (1, 7). Disruption of RDH12, the IS-localized dehydrogenase implicated in preventing cytotoxic all-*trans*-retinal accumulation, shifted the RE profile toward all-*trans*-REs, paralleling human IRD phenotypes associated with pathological RDH12 variants, which are characterized by heightened sensitivity to retinaldehyde toxicity (8). Together, these findings reinforce the concept that photoreceptor IS function as active retinoid-processing hubs, balancing chromophore supply with detoxification.

Perhaps the most striking observation in this study was the pattern of retinoid trapping in MG. When GFAP-driven LRAT was introduced via adeno-associated virus, promoting selective expression in MGs, REs appeared throughout MG processes with a nearly exclusive all-trans configuration. This suggests that MGs encounter abundant all-*trans*-retinol (likely originating from photoreceptors or circulation) but far less 11-*cis*-retinol than previously proposed in models of MG-based chromophore regeneration. This finding challenges the extent to which MGs contribute to cone-specific chromophore regeneration in mouse retina, a mechanism that is well characterized in nonmammalian species like zebrafish but remains less clear in mammals. Instead, Engfer et al. demonstrated that murine MGs appear tuned more toward detoxification or buffering of all-*trans*-retinoids rather than participating in isomerization or 11-cis supply (1). Notably, the investigators identified robust endogenous LRAT expression in human (but not mouse) MGs in single-cell transcriptomic datasets, with a regional bias toward peripheral MG outside of the macula region. This species difference invites reconsideration of human MG contributions to peripheral cone support, chromophore recycling, and detoxification. It also underscores the importance of corroborative studies using human models when evaluating MG-targeted therapies.

Using RGC-specific LRAT expression, the investigators demonstrated that RGCs formed distinct, acyl chain–specific REs, indicating access to all-*trans*-retinol and, under prolonged dark adaptation, 11-*cis*-retinol. This observation has implications for melanopsin-based phototransduction in intrinsically photosensitive RGCs (ipRGCs), whose chromophore requirements remain incompletely defined. The finding that RGCs are exposed to both retinoid isomers supports hypotheses that ipRGCs rely on, at least in part, local retinoid sources.

## Clinical and translational implications and conclusions

IRDs comprise a genetically and clinically heterogeneous group of single-gene disorders affecting approximately 1 in 2,000–3,000 individuals worldwide and represent a major cause of irreversible vision loss (9). Studies of IRDs arising from disruption of the visual cycle have been particularly informative, as they directly link defects in retinoid metabolism to photoreceptor dysfunction and degeneration. Individuals with visual cycle IRDs can experience early and progressive night blindness, constriction of visual fields, and impaired contrast sensitivity, with substantial effects on mobility, independence, and quality of life even when visual acuity remains relatively preserved (10). At a molecular level, pathogenic variants in genes encoding visual cycle enzymes and retinoid-binding proteins (e.g., RPE65, LRAT, RDH12, and CRALBP) impair chromophore regeneration, promote accumulation of toxic retinoid intermediates, and induce photoreceptor stress and cell death (11–14). These processes are mechanistically distinct from primary phototransduction defects, yet they converge on shared pathways of retinal degeneration, underscoring a central role for retinoid flux in maintaining photoreceptor integrity (15).

Insights into visual cycle biology have directly informed therapeutic development, culminating in gene augmentation strategies for RPE65-associated disease and motivating parallel approaches targeting other components of the retinoid cycle (16–19). However, variability in therapeutic durability and efficacy highlights persistent gaps in understanding how retinoids are trafficked, buffered, and detoxified within specific retinal cell types and disease stages (15, 20). Emerging optogenetic and gene-based strategies further underscore the need to define retinoid availability and buffering within inner retinal neurons, particularly in late-stage degeneration (21). Complementary advances in human retinal imaging and disease modeling have begun to refine this understanding. High-resolution in vivo imaging studies have revealed cell type–specific patterns of photoreceptor loss and structural remodeling in IRDs, enabling correlation of genotype, retinal structure, and functional decline (22, 23). In parallel, human pluripotent stem cell–derived retinal organoids and explant systems have provided experimentally tractable platforms to interrogate visual cycle gene function, retinoid metabolism, and therapeutic response in a human genetic context (24). Together, these approaches underscore the importance of integrating spatially resolved retinoid biology with human-relevant models and imaging biomarkers.

In this context, direct visualization of retinoid distribution across neural retinal compartments, as enabled by the approach described by Engfer et al., provides critical mechanistic insight into visual cycle IRDs and offers a rational framework for refining gene-, cell-, and optogenetic therapies aimed at preserving or restoring vision (20, 25). The ability to map retinoid distribution in vivo has several implications for retinal disease. First, by identifying the exact compartments that encounter all-*trans*-retinol, this study clarifies where toxic aldehydes may accumulate in disease and highlights potential nodes for therapeutic intervention. Second, knowing the spatial availability of retinoids will improve design of LRAT, RPE65, or RDH12 gene therapy strategies by predicting substrate access in treated cells. Furthermore, the discovery of LRAT expression in human MG but not murine MG underscores caution when extrapolating chromophore pathways across species. Finally, RE distributions identified in Engfer et al.’s study may guide interpretation of autofluorescence imaging, which detects retinoid adducts such as bisretinoids.

In conclusion, Engfer et al. have presented a powerful new approach that resolves the spatial dynamics of retinoids in vivo with unprecedented clarity. Their findings reinforce and refine the canonical biochemical map of the visual cycle while revealing unexpected species differences and new cellular participants. The implications for understanding IRDs and designing targeted interventions are substantial. Their study highlights the importance of merging classical visual cycle biochemistry with modern genetic and imaging tools, a combination that promises to improve the resolution of our understanding of human retinal physiology and pathology.

## Funding support

This work is the result of NIH funding, in whole or in part, and is subject to the NIH Public Access Policy. Through acceptance of this federal funding, the NIH has been given a right to make the work publicly available in PubMed Central.

- National Eye Institute, NIH grants R01EY029750, EY035889, and EY032104 to AS and R01EY034123 to AM.
- Research to Prevent Blindness Inc. Special Scholar Award to AS.
- Unrestricted grants to the Wilmer Eye Institute, Johns Hopkins School of Medicine.
- Norman Raab Foundation to AS.
- Branna and Irving Sisenwein Professorship in Ophthalmology to AS.

## Figures and Tables

**Figure 1:**
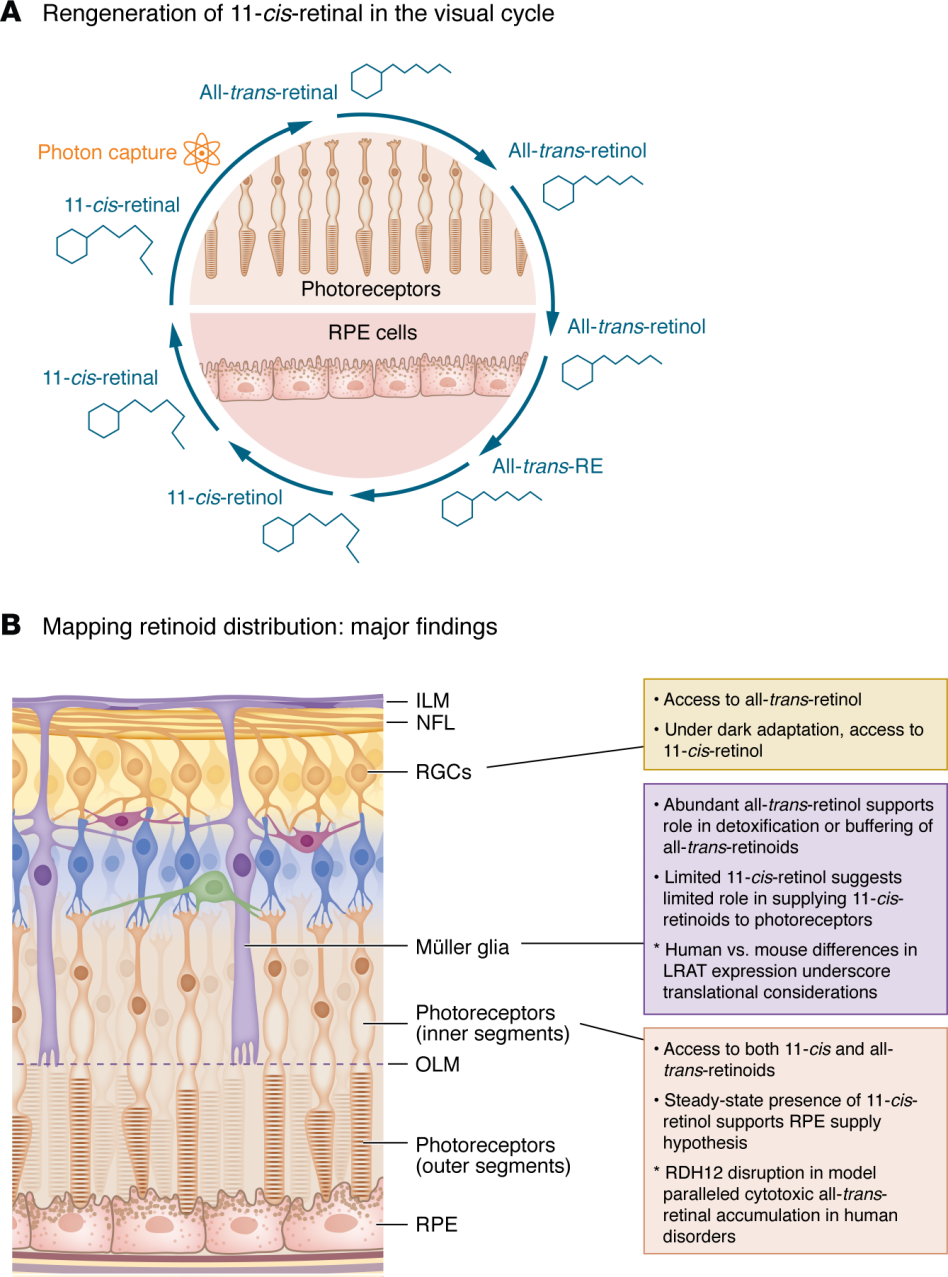
Retinoid regeneration and distribution in the retina. (**A**) Brief summary of the visual cycle. Upon photon capture, 11-*cis*-retinal is converted to trans-retinoids and back to 11-*cis*-retinoids. This process, which occurs mainly in photoreceptors and RPE cells, is essential for recycling 11-*cis*-retinal and maintaining its levels in photoreceptors. (**B**) Engfer et al. (1) leveraged cell-type expression of LRAT to trap fluorescently detectable REs in specific retinal compartments. Notable observations for each cell type are described. Together, these insights provide a framework for improving a variety of therapies aimed at vision restoration. ILM, internal limiting membrane; NFL, nerve fiber layer; OLM, outer limiting membrane.

## Version 1 02/02/2026

Electronic publication

## References

[1] Engfer ZJ (2026). Retinol tracing within murine neural retina reveals cell type–specific retinol transport and distribution. J Clin Invest.

[2] Travis GH (2007). Diseases caused by defects in the visual cycle: retinoids as potential therapeutic agents. Annu Rev Pharmacol Toxicol.

[3] Palczewski K (2012). Chemistry and biology of vision. J Biol Chem.

[4] Kiser PD, Palczewski K (2016). Retinoids and retinal diseases. Annu Rev Vis Sci.

[5] Redmond TM et al (1998). Rpe65 is necessary for production of 11-cis-vitamin A in the retinal visual cycle. Nat Genet.

[6] Swaroop A (2010). Transcriptional regulation of photoreceptor development and homeostasis in the mammalian retina. Nat Rev Neurosci.

[7] Koutalos Y, Yau KW (1996). Regulation of sensitivity in vertebrate rod photoreceptors by calcium. Trends Neurosci.

[8] Maeda A (2009). Retinal disease caused by accumulation of toxic retinoid byproducts. J Biol Chem.

[9] Pierce EA, Bennett J (2015). The status of RPE65 gene therapy trials: safety and efficacy. Cold Spring Harb Perspect Med.

[10] Jayasundera T (2010). Impact of inherited retinal degenerations on quality of life and functional vision. Ophthalmology.

[11] Redmond TM (1998). Rpe65 is necessary for production of 11-cis-vitamin A in the retinal visual cycle. Nat Genet.

[12] Ruiz A (1999). Molecular and biochemical characterization of mutations in the human lecithin retinol acyltransferase (LRAT) gene. Hum Mol Genet.

[13] Janecke AR (2004). Mutations in RDH12 encoding a photoreceptor cell retinol dehydrogenase cause childhood-onset severe retinal dystrophy. Nat Genet.

[14] Saari JC (2001). Visual cycle impairment in cellular retinaldehyde binding protein (CRALBP) knockout mice results in delayed dark adaptation. Neuron.

[15] Duncan JL (2018). Inherited retinal degenerations: current landscape and knowledge gaps. Transl Vis Sci Technol.

[16] Bennett J (2012). AAV2 gene therapy readministration in three adults with congenital blindness. Sci Transl Med.

[17] Pennesi ME (2018). Results at 5 years after gene therapy for RPE65-deficient retinal dystrophy. Hum Gene Ther.

[18] Tsang SH, Sharma T (2018). Retinitis pigmentosa (Non-syndromic). Adv Exp Med Biol.

[19] Comander J (2013). Visual function in patients with RPE65-associated Leber congenital amaurosis. Ophthalmology.

[20] Van Gelder RN, Sherwood MB (2020). Acute effects of intraocular pressure-induced changes in schlemm’s canal morphology on outflow facility in healthy human eyes. Invest Ophthalmol Vis Sci.

[21] Sahel JA (2021). Partial recovery of visual function in a blind patient after optogenetic therapy. Nat Med.

[22] Carroll J (2012). High-resolution retinal imaging reveals photoreceptor loss patterns in inherited retinal degeneration. Proc Natl Acad Sci U S A.

[23] Nagiel A (2016). High-resolution imaging with adaptive optics in patients with inherited retinal degeneration. Invest Ophthalmol Vis Sci.

[24] Gamm DM (2014). A novel serum-free method for generating human retinal organoids with functional photoreceptors. Nat Biotechnol.

[25] Flannery JG, Byrne LC (2018). Gene therapy approaches for inherited retinal degenerations. Cold Spring Harb Perspect Med.

